# Supplementary figures and images for: A toxin/antitoxin system targeting the replication sliding-clamp induces competence in Streptococcus pneumoniae
Source: PLoS Genet. 2025 Dec 29;21(12):e1011863. doi: 10.1371/journal.pgen.1011863 (PMC12795458; doi:10.1371/journal.pgen.1011863)

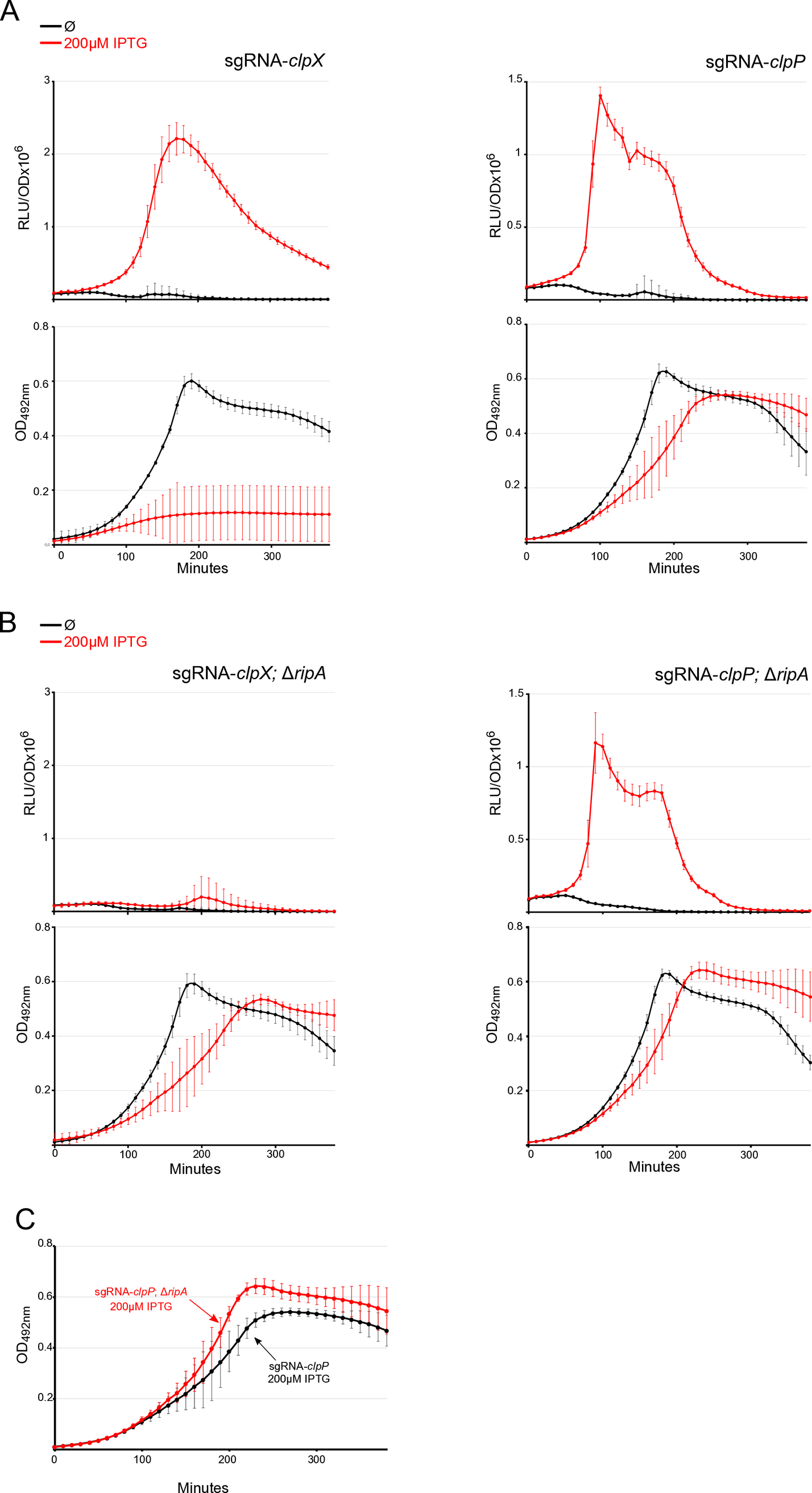

Supplement: S1 Fig — A. comCDE expression was monitored in strains carrying CRISPRi guide RNA targeting clpX, R4993 (left) or clpP R5203 (right). Cells were grown in C + Y medium at 37°C with or without IPTG, as shown in the colour key. Culture was initiated 100 minutes prior to the first measurement at time 0. comCDE expression values are expressed in relative light units per OD (RLU/OD) in the top panel and the corresponding growth curves are reported as OD492nm in the bottom panel. Data represented as Mean ± standard deviation of triplicate repeats. B. Identical to A in ΔripA strains carrying CRISPRi guide RNA targeting respectively clpX, R4995 (left) or clpP, R5250 (right). Data represented as Mean ± standard deviation of triplicate repeats. C. Comparison of the growth curves of ClpP-depleted strains in the presence or absence of the ripA gene, plotted on a linear y-axis. Red: ΔripA; Black: ripA wild-type. (TIF) [file pgen.1011863.s001.tif]

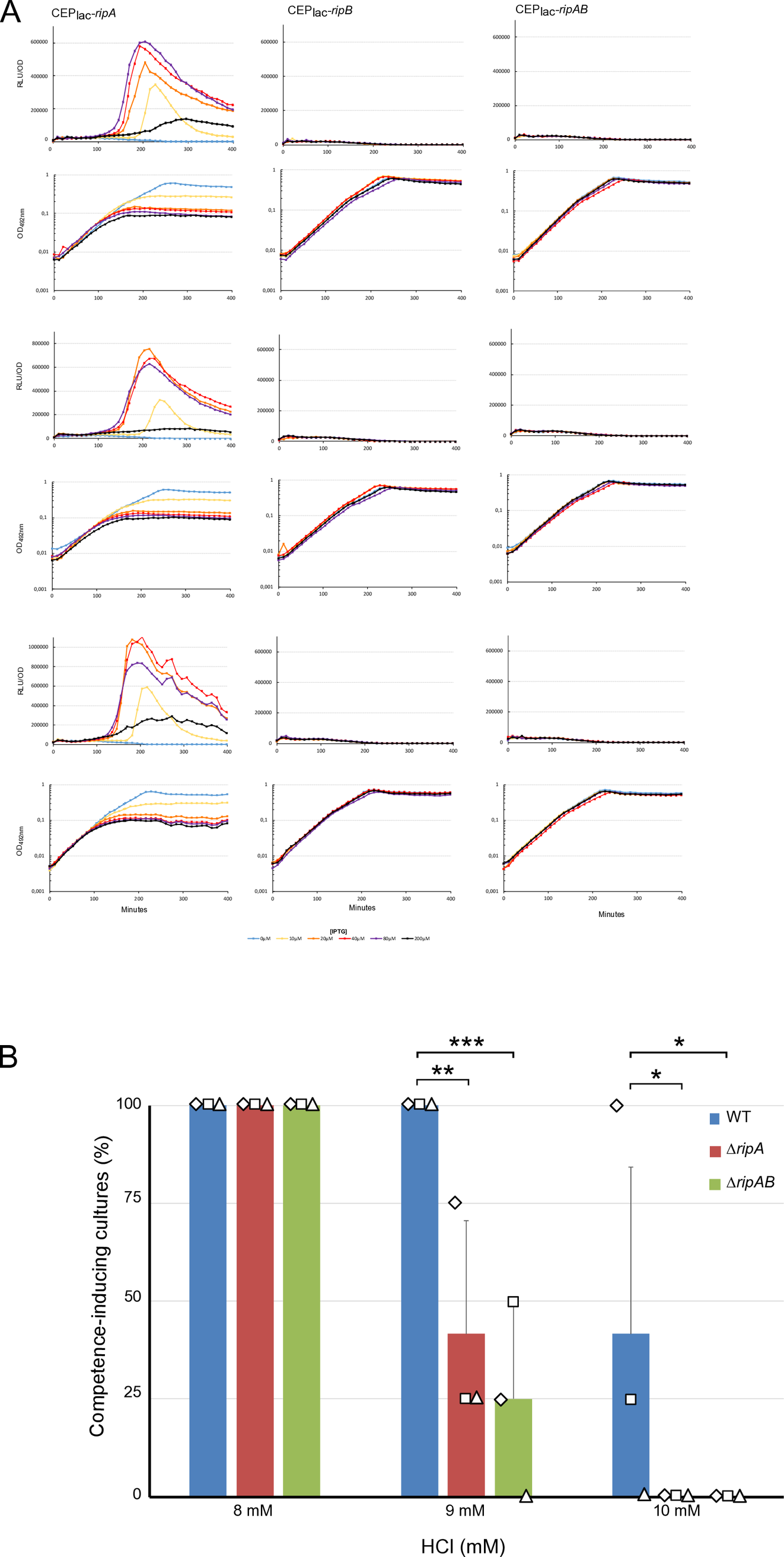

Supplement: S2 Fig — A. Replicates of Fig 2B. comCDE expression was monitored in strains expressing ripA (R5139), ripB (R5138) or ripA-ripB (R5140) under the control of an IPTG inducible promotor. Cells were grown in C + Y medium at 37°C with increasing concentration of IPTG from the first measurement at time 0. Top panels: luciferase activity expressed in relative light units per OD (RLU/OD). Bottom panels: corresponding growth curves. B. Deletion of the ripA or ripAB locus reduces competence induction efficiency. R825, R4422, and R4423 strains were cultured in C + Y medium under conditions near the competence induction threshold (mild medium acidification). For each experiment, four independent cultures per strain were monitored, and the proportion of cultures undergoing competence induction was quantified. The experiment was performed three times on separate days. The standard deviation represents the variability in competence induction frequencies across the three independent experiments. (TIF) [file pgen.1011863.s002.tif]

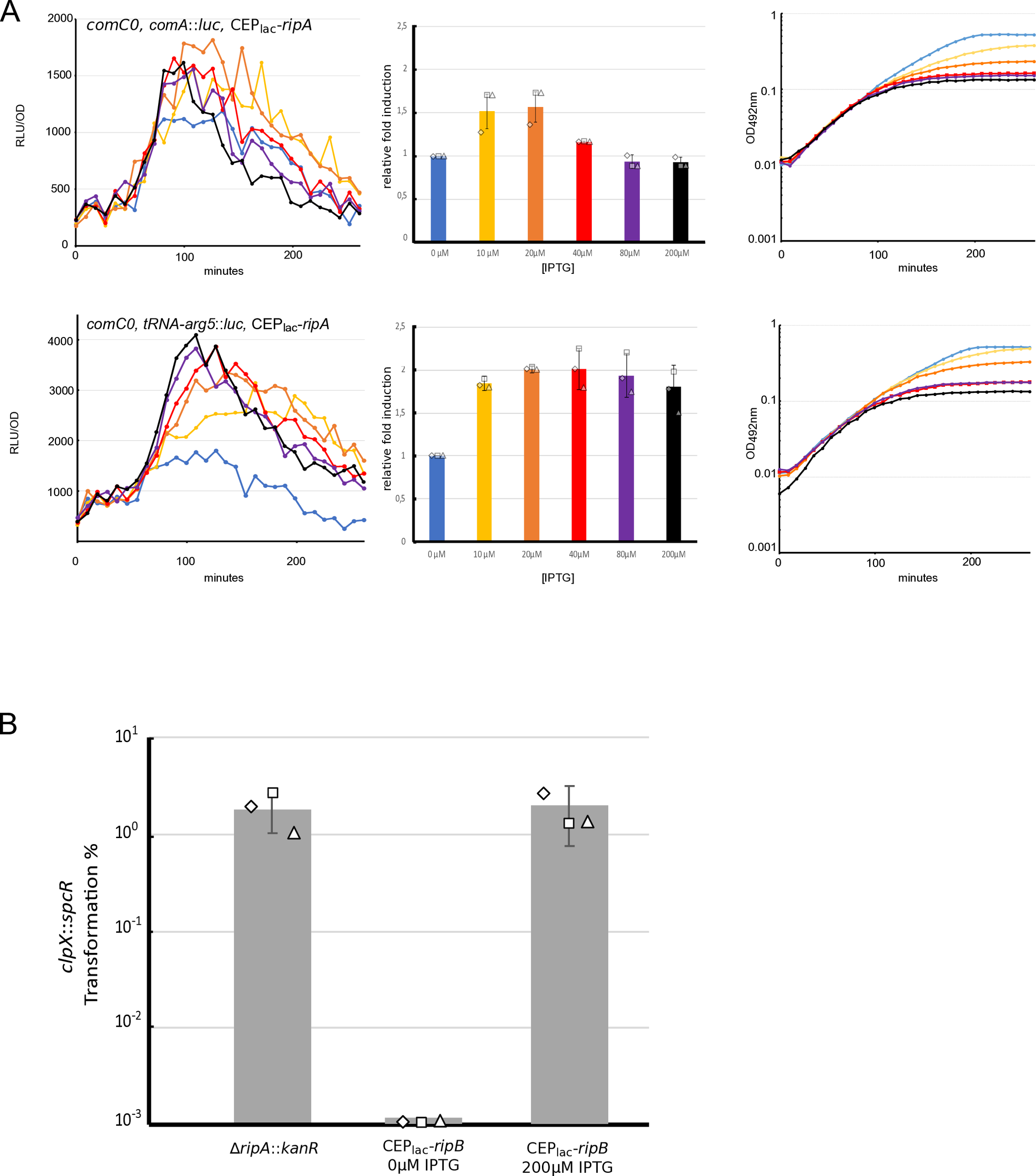

Supplement: S3 Fig — A. Induction of basal transcription levels of comA and tRNA-arg5. comC0-derived strains producing RipA under an IPTG-inducible promoter were grown in the presence of increasing IPTG concentrations. comA (R5572) and tRNA-arg5 (R5571) transcription levels were monitored through luciferase activity (left panels). For clarity, only one representative experiment is shown; however, the areas under the curves were quantified from three independent experiments and are presented as bar graphs with their respective standard deviations as shown in the middle panels. Right panels: corresponding growth curves. B. Overexpression of RipB compensates for the loss of clpX. Strain R5565 overexpressing ripB under the control of an IPTG-inducible promoter was transformed with a clpX deletion PCR fragment and then plated in the presence or absence of 200 µM IPTG. R4796 strain, deleted for ripA, was used as a positive control for transformation (n = 3). (TIF) [file pgen.1011863.s003.tif]

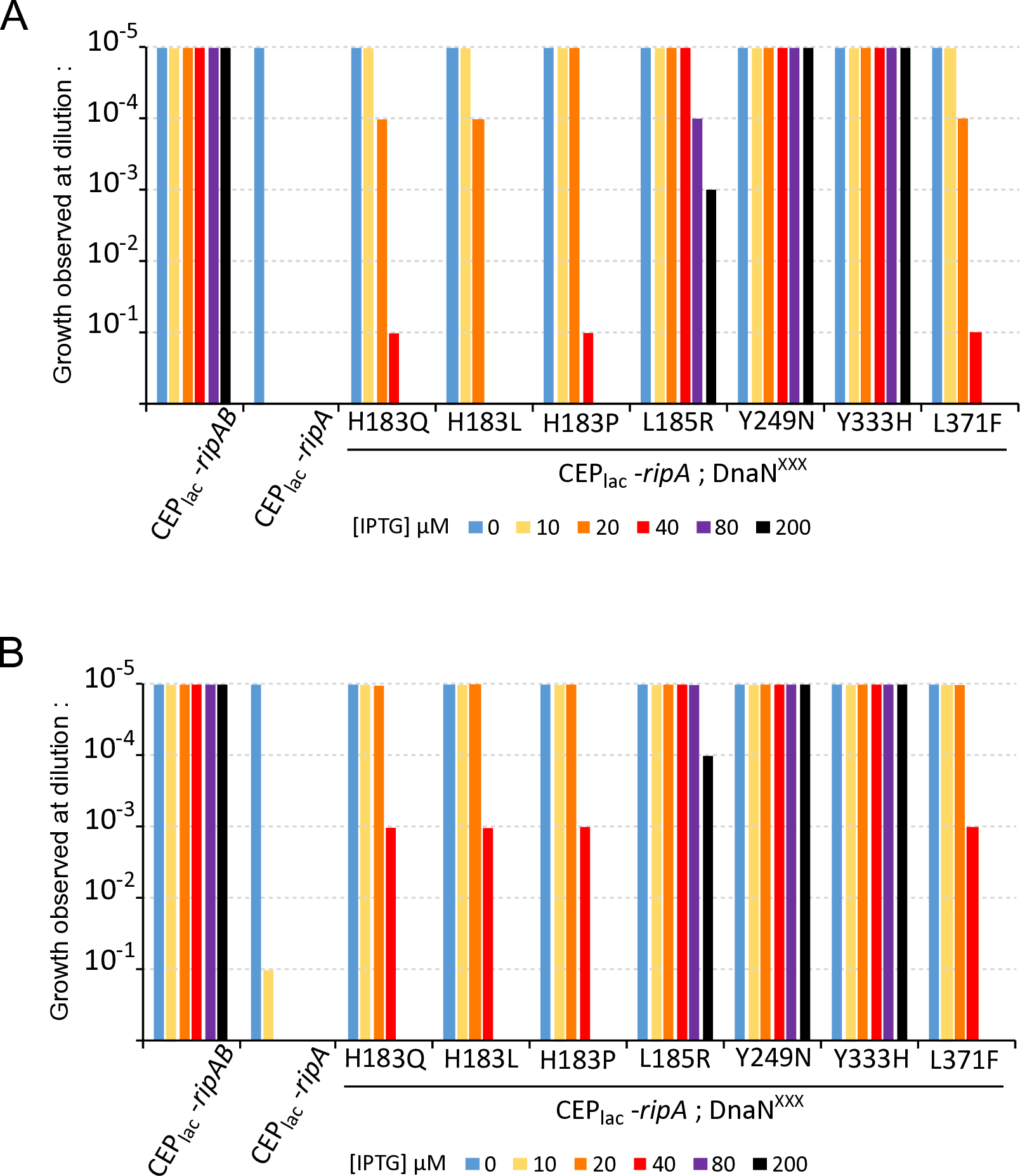

Supplement: S4 Fig — Efficiency of plating of strains expressing ripA under the control of an IPTG inducible promotor in a dnaN wild type genetic background (R5139) or in a DnaN suppressor mutations genetic background (R5165 to R5171). The strain expressing ripA-ripB under the control of IPTG was used as a growth control (R5140). Cells were plated on CAT agar supplemented with 4% horse blood containing different concentrations of IPTG. For clarity, each single spot test is presented as a histogram. (TIF) [file pgen.1011863.s004.tif]

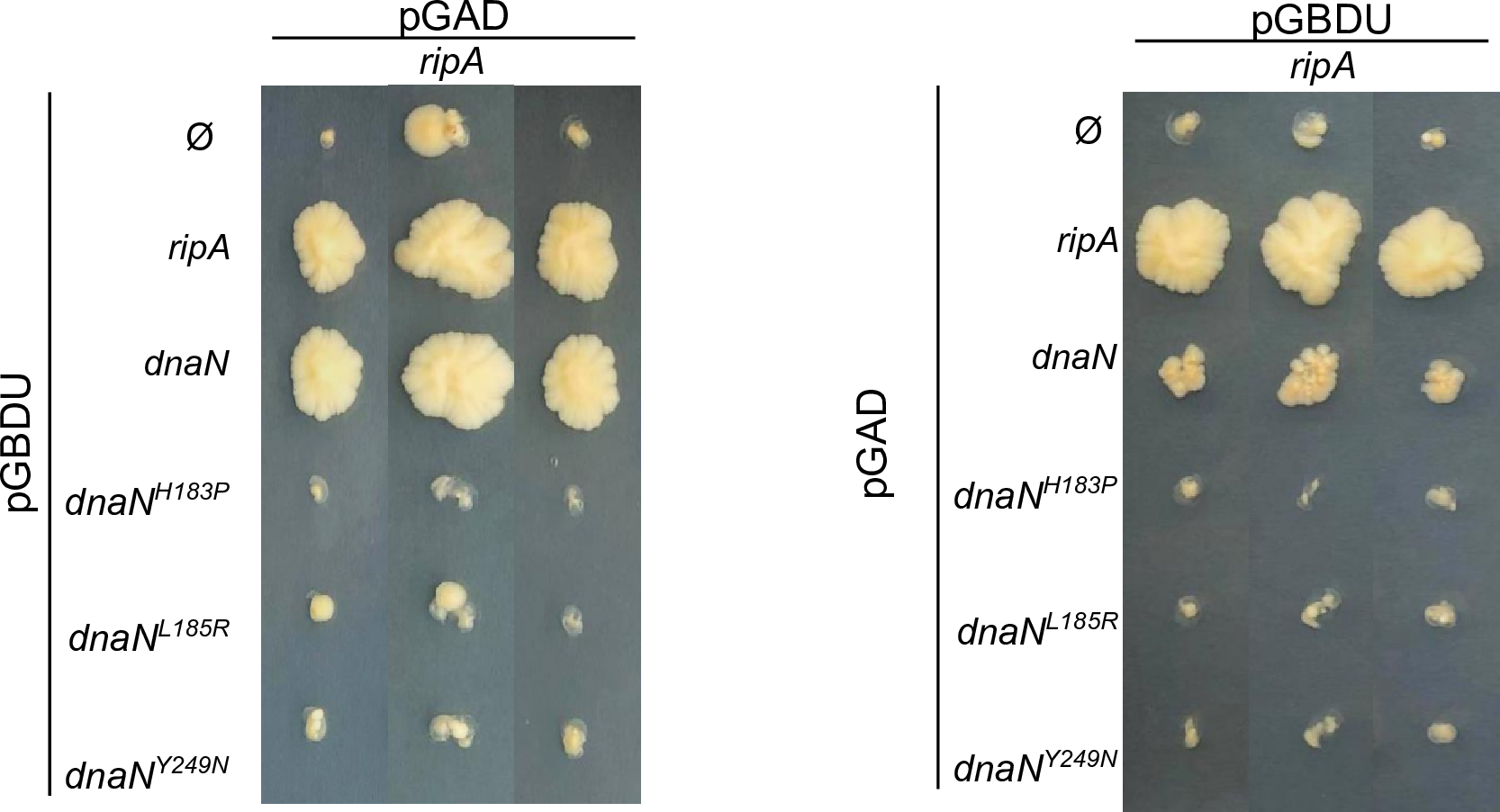

Supplement: S5 Fig — Three independent yeast two-hybrid experiments were performed in triplicate, testing the interaction in both possible orientations. (TIF) [file pgen.1011863.s005.tif]

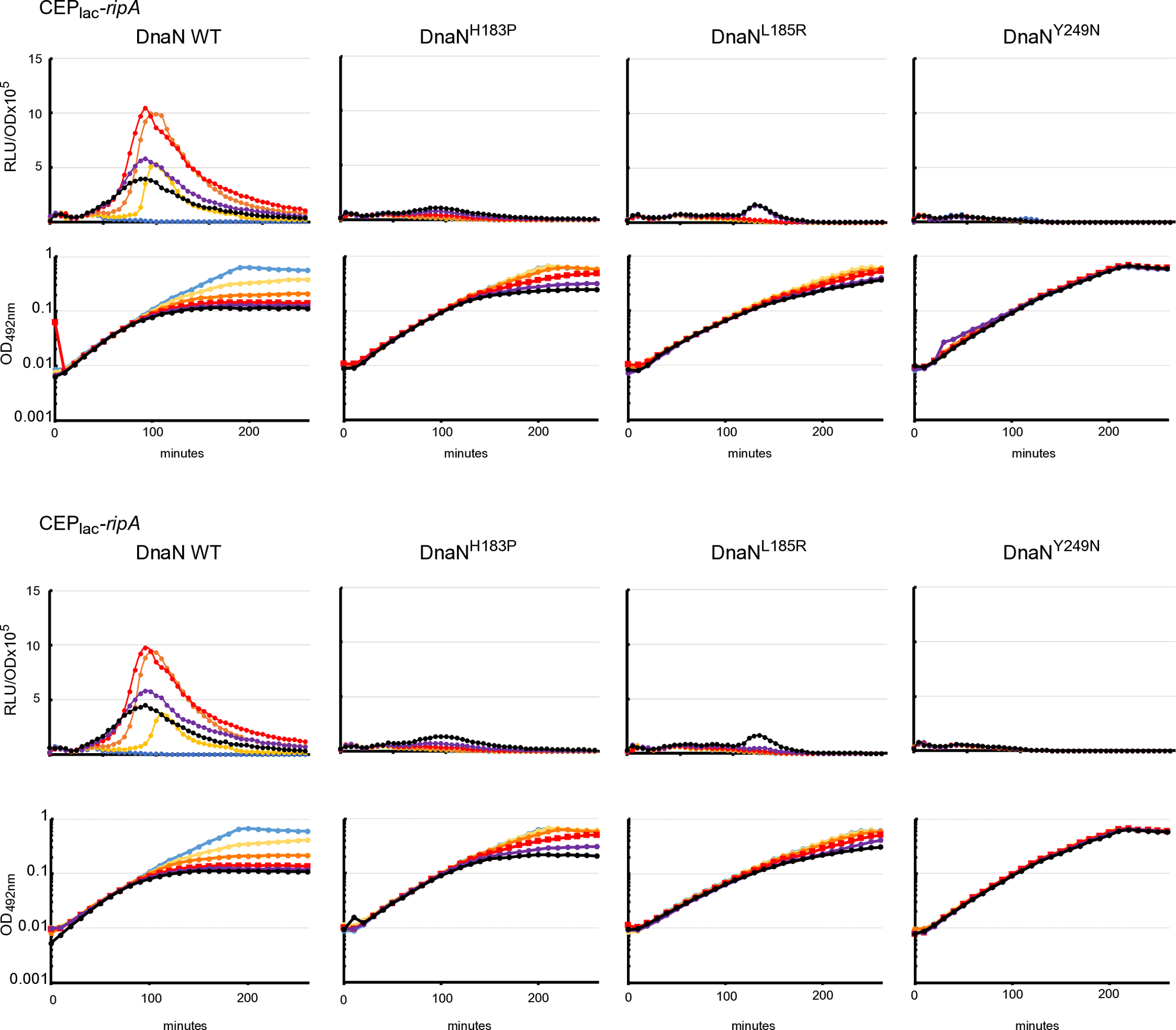

Supplement: S6 Fig — Two replicates of experiments displayed Fig 6. comCDE expression was monitored in strains expressing ripA under the control of an IPTG inducible promotor in a wild type dnaN genetic background (R5138) or in strains carrying suppressive alleles of dnaN, H183P (R5167), L185R (R5168), Y249N (R5169), conferring respectively weak, medium and strong suppressive phenotype. Cells were grown in C + Y medium at 37°C with increasing amounts of IPTG from the first measurement at time 0. Top panels: luciferase activity expressed in relative light units per OD (RLU/OD). Bottom panels: corresponding growth curves. (TIF) [file pgen.1011863.s006.tif]

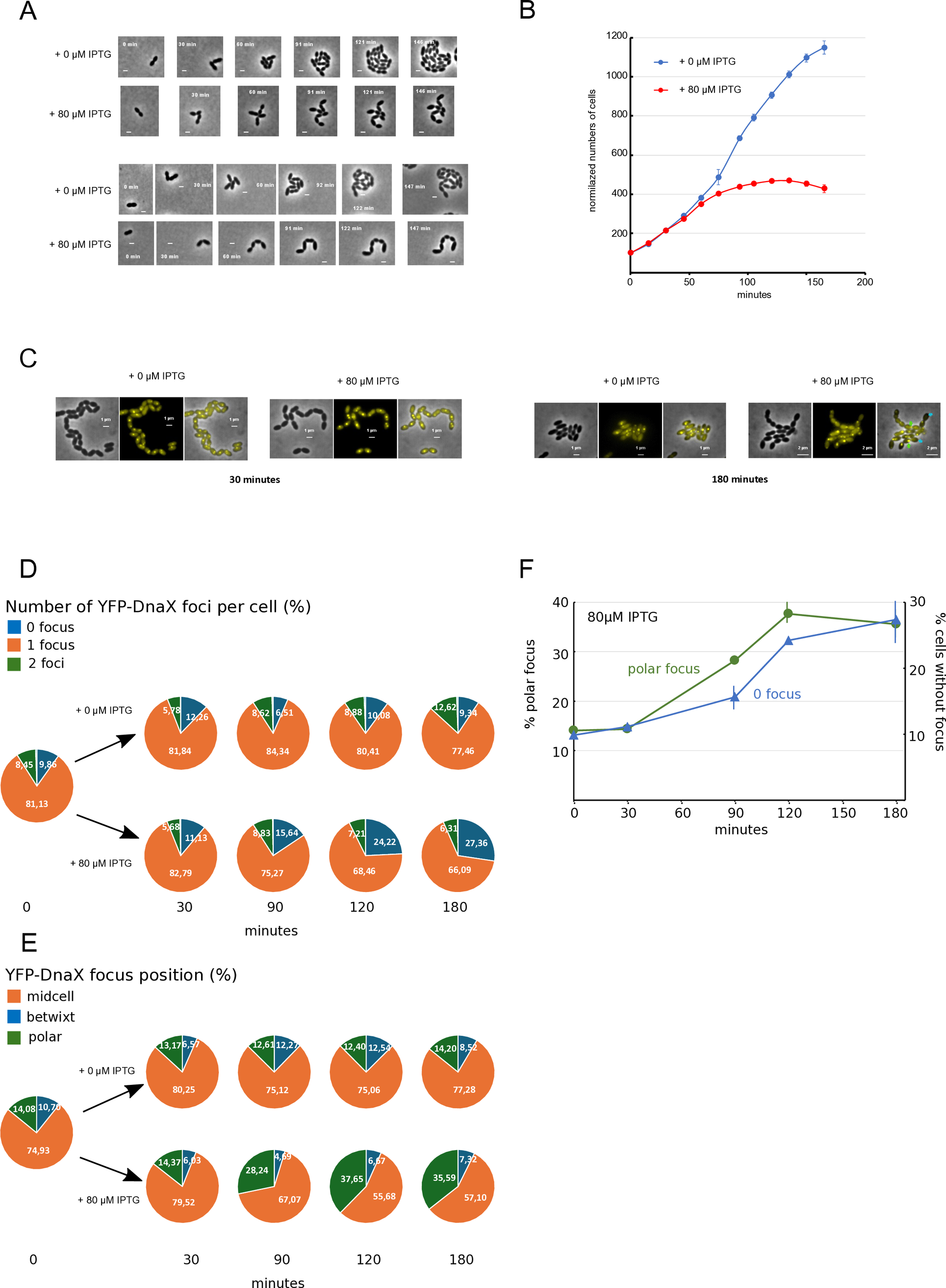

Supplement: S7 Fig — A. Phase contrast time-lapses of the strain expressing ripA under the control of an IPTG inducible promotor (R5139) without or with 80µM of IPTG (respectively top and bottom panels). Two independent experiments. B. Microscopy-based cell enumeration showed in A. Means of two independent experiments are shown, together with their standard deviations. The initial cell number at T0 was normalized to 100. Actual initial cell counts were: without IPTG, n = 398 and 372; with IPTG, n = 364 and n = 475. C. Representative microscopy images of the R5260, expressing YFP-DnaX and carrying Plac-ripA, in the presence or absence of IPTG. Blue arrows; cells without focus, green arrows; cells with polar focus. D. Quantification of the number of YFP-DnaX fluorescent foci per cell in the R5260 grown with or without IPTG. Number of cells analysed at T0: n = 710, T30: n = 720 + 962, T90: n = 518 + 1675, T120: n = 2382 + 819, T180; n = 1312 + 1130. With IPTG, T30: n = 766 + 1390, T90: n = 2542 + 1442, T120: n = 3119 + 1266, T180: n = 3843 + 1889. Results without IPTG; 0 focus T30: 12.26 ± 3.98, T90: 6.51 ± 2.53, T120: 10.08 ± 5.77, T180: 9.34 ± 2.32. 1 focus T30: 81.84 ± 0.34, T90: 84.34 ± 2.98, T120: 80.41 ± 0.10, T190: 77.46 ± 1.91. 2 foci: T30: 5.78 ± 3.61, T90: 8.62 ± 0.37, T120: 8.88 ± 4.78, T180: 12.62 ± 0.17. Results with IPTG; 0 focus: T30: 11.13 ± 0.23, T90: 15.64 ± 0.9, T120: 24.22 ± 0.15, T180: 27.36 ± 6.08. 1 focus: T30: 82.79 ± 3.17, T90: 75.27 ± 2.31, T120: 68.46 ± 1.31, T180: 66.09 ± 4.31. 2 foci: T30: 5.68 ± 3.05, T90: 8.83 ± 0.86, T120: 7.21 ± 1.31, T180: 6.31 ± 1.67. E. Localization of YFP-DnaX fluorescent foci in R5260 under IPTG-induced and non-induced conditions. Number of foci localized at T0: n = 710, T30: n = 714 + 850, T90: n = 527 + 1752, T120: n = 2592 + 749, T180; n = 1402 + 1154. With IPTG, T30: n = 752 + 1288, T90: n = 2314 + 1390, T120: n = 2592 + 1061, T180: n = 3270 + 1394. Results without IPTG; midcell: T30: 80.25 ± 0.19, T90: 75.12 ± 2.18, T120: 75.06 ± [file pgen.1011863.s007.tif]
