# Supplementary material for: A toxin/antitoxin system targeting the replication sliding-clamp induces competence in Streptococcus pneumoniae
Source: PLoS Genet. 2025 Dec 29;21(12):e1011863. doi: 10.1371/journal.pgen.1011863 (PMC12795458; doi:10.1371/journal.pgen.1011863)
Supplement: S1 Table — (DOCX) [file pgen.1011863.s008.docx]

**Supporting Tables:**

**S1 Table: Strains.**

| Strain | Genotype/relevant feature | References |
| --- | --- | --- |
| R800 |  |  |
| R825 | *comC, comC*::[*luc, ermAM*] | (1) |
| R1501 | *ComC0* | (2) |
| R3833 | *ComC2D1,* CEP_lac_::*dprA, dprA*::*aad9* | (3) |
| R4422 | *comC, comC*::[*luc, ermAM*]*,* ∆*ripA*::*aphA-3* | this study |
| R4423 | *comC, comC*::[*luc, ermAM*]*,* ∆*ripA*::*aphA-3,* ∆*ripB*::*aad9* | this study |
| R4631 | *comC0, ssbB-luc CEP_mal_-yfp-dnaX, CEPII_lac_-**dprA-mturquoise, dprA*::*aad9* | (4) |
| R4796 | *ripA*::*aphA-3* | this study |
| R4856 | *comC0*, *dprA-lgbit*, *dnaX-smbit* | (4) |
| R4993 | *comC, comC*::[*luc, ermAM*]*,* PF6*-lacI,* P_lac_ *dCas9,* P3*-*sgRNA*-clpX* | this study |
| R4995 | *comC, comC*::[*luc, ermAM*]*,* PF6*-lacI,* P_lac_ *dCas9,* P3*-*sgRNA*-clpX,* ∆*ripA*::*aad9* | this study |
| R5085 | *comC0, ∆ripAB*::*aad9,* PF6-*lacI* | this study |
| R5086 | *comC0, ∆ripAB*::*aad9,* PF6-*lacI,* CEP_lac_::*ripA* | this study |
| R5089 | *comC0, comC*::[*luc, ermAM*]*,∆ripAB*::*aad9,*PF6-*lacI,* CEP_lac_::*ripA* | this study |
| R5138 | *comC, comC*::[*luc, ermAM*]*,* ∆*ripAB*::*aad9,*PF6*-lacI,* CEP_lac_::*ripB* | this study |
| R5139 | *comC, comC*::[*luc, ermAM*]*,* ∆*ripA*::*aad9,*PF6*-lacI,* CEP_lac_::*ripA* | this study |
| R5140 | *comC, comC*::[*luc, ermAM*]*,* ∆*ripAB*::*aad9,*PF6*-lacI,* CEP_lac_::*ripAB* | this study |
| R5165 | *comC, comC*::[*luc, ermAM*]*,* ∆*ripAB*::*aad9,*PF6*-lacI,* CEP_lac_::*ripA, dnaN^H183Q^* | this study |
| R5166 | *comC, comC*::[*luc, ermAM*]*,* ∆*ripAB*::*aad9,*PF6*-lacI,* CEP_lac_::*ripA, dnaN^H183L^* | this study |
| R5167 | *comC, comC*::[*luc, ermAM*]*,* ∆*ripAB*::*aad9,*PF6*-lacI,* CEP_lac_::*ripA, dnaN^H183P^* | this study |
| R5168 | *comC, comC*::[*luc, ermAM*]*,* ∆*ripAB*::*aad9,*PF6*-lacI,* CEP_lac_::*ripA, dnaN^L185R^* | this study |
| R5169 | *comC, comC*::[*luc, ermAM*]*,* ∆*ripAB*::*aad9,*PF6*-lacI,* CEP_lac_::*ripA, dnaN^Y249N^* | this study |
| R5170 | *comC, comC*::[*luc, ermAM*]*,* ∆*ripAB*::*aad9,*PF6*-lacI,* CEP_lac_::*ripA, dnaN^Y333H^* | this study |
| R5171 | *comC, comC*::[*luc, ermAM*]*,* ∆*ripAB*::*aad9,*PF6*-lacI,* CEP_lac_::*ripA, dnaN^L371F^* | this study |
| R5198 | Δ*ripAB*::*aad9R,* PF6*-lacI* | this study |
| R5203 | *comC*::[*luc, ermAM*]*, PF6-lacI,* P_lac_*-dCas9,* P3*-*sgRNA*-clpP* | this study |
| R5204 | ∆*ripAB*::*aad9,*PF6-lacI*,* CEP*_lac_*::*ripA* | this study |
| R5239 | ∆*ripAB*::*aad9,*PF6-lacI*,* CEPII_lac_::*ripA* | this study |
| R5250 | *comC*::[*luc, ermAM*]*,* PF6-*lacI,* P_lac_*-dCas9,*P3*-*sgRNA*-clpX,* ∆*ripA*::*aad9* | this study |
| R5259 | ∆*ripAB*::*aad9,*PF6*-lacI,* CEPII_lac_::*ripA,* CEP_lac_::*ripB aphA-3* | this study |
| R5260 | ∆*ripAB*::*aad9,*PF6*-lacI,* CEPII_lac_::*ripA*, CEP_mal_::*YFP-dnaX* | this study |
| R5352 | *comC0,* ∆*ripAB*::*aad9,* PF6-lacI*,* CEP_lac_::*lgbit-ripA* | this study |
| R5370 | *comC0,* ∆*ripAB*::*aad9,* PF6-lacI*,* CEPII_lac_::*smbit-ripA* | this study |
| R5380 | *comC0,* ∆*ripAB*::*aad9,* PF6-lacI*,* CEP_lac_::*Lgbit,* CEPII_lac_::*smbit-ripA* | this study |
| R5381 | *comC0,* ∆*ripAB*::*aad9,* PF6-*lacI,* CEP*_lac_*::*lgbit-ripA aphA-3,* CEPII_lac_::*smbit-dnaN* | this study |
| R5565 | CEP_lac_::*ripB* | this study |
| R5566 | *comC0,* ∆*ripAB*::*aad9,* PF6-*lacI,* CEP_lac_::*lgbit-ripA aphA-3,* CEPII_lac_::*smbit-dnaN^H18PL^* | this study |
| R5567 | *comC0, ∆ripAB*::*aad9,* PF6*-lacI,* CEP_lac_::*lgbit-ripA aphA-3,* CEPII_lac_::*smbit-dnaN^L185R^* | this study |
| R5568 | *comC0, ∆ripAB*::*aad9,* PF6*-lacI,* CEP_lac_::*lgbit-ripA aphA-3,* CEPII_lac_::*smbit-dnaN^Y249N^* | this study |
| R5570 | *comC0, ∆ripAB*::*aad9,* PF6*-lacI,* CEP_lac_::*lgbit,* CEPII_lac_::*smbit-dnaN* | this study |
| R5571 | *comC0, tRNAarg5*::[*luc, cat*], ∆*ripAB*::*aad9,* PF6-*lacI,* CEP_lac_::*ripA* | this study & (5) |
| R5572 | *comC0, comA*::[*luc, ermAM*]*,* ∆*ripAB*::*aad9,* PF6-*lacI,* CEP_lac_::*ripA* | this study & (6) |

Resistance genes*: aad9*: [spc^R^], *aphA-3*: [kan^R^], *ermAM*: [ery^R^], *cat*: [Cm^R^]

1. Bergé M, Moscoso M, Prudhomme M, Martin B, Claverys J-P. Uptake of transforming DNA in Gram-positive bacteria: a view from Streptococcus pneumoniae. Mol Microbiol. 2002;45: 411–421.
2. Dagkessamanskaia A, Moscoso M, Hénard V, Guiral S, Overweg K, Reuter M, et al. Interconnection of competence, stress and CiaR regulons in Streptococcus pneumoniae: competence triggers stationary phase autolysis of ciaR mutant cells. Mol Microbiol. 2004;51: 1071–1086.
3. Johnston C, Mortier-Barriere I, Khemici V, Polard P. Fine-tuning cellular levels of DprA ensures transformant fitness in the human pathogen Streptococcus pneumoniae. Mol Microbiol. 2018;109: 663–675.
4. Johnston CHG, Hope R, Soulet A-L, Dewailly M, De Lemos D, Polard P. The RecA-directed recombination pathway of natural transformation initiates at chromosomal replication forks in the pneumococcus. Proc Natl Acad Sci U S A. 2023;120.
5. Martin B, Granadel C, Campo N, Hénard V, Prudhomme M, Claverys J-P. Expression and maintenance of ComD-ComE, the two-component signal-transduction system that controls competence of Streptococcus pneumoniae. Mol Microbiol. 2010;75: 1513–1528.
6. Maziero M, Lane D, Polard P, Bergé M. Fever-like temperature bursts promote competence development via an HtrA-dependent pathway in Streptococcus pneumoniae. PLoS Genet. 2023;19
