# Supplementary material for: A toxin/antitoxin system targeting the replication sliding-clamp induces competence in Streptococcus pneumoniae
Source: PLoS Genet. 2025 Dec 29;21(12):e1011863. doi: 10.1371/journal.pgen.1011863 (PMC12795458; doi:10.1371/journal.pgen.1011863)
Supplement: S2 Table — (DOCX) [file pgen.1011863.s009.docx]

**S2 Table: Primers used in this study**

|  | | |  |
| --- | --- | --- | --- |
| **Y2H** | | | |
| MM28 | TTCCCCGGGGGATCCATGATTCATTTTTCAATTAATAAAAATTTATTTC | *dnaN* |  |
| MM29 | CAGGTCGACATCGATTTAATTTGTACGAACTGGTGTAATGAGC | *dnaN* |  |
| MM30 | TTCCCCGGGGGATCCATGTTGAATCAGGATCTCTTTGATTCGC | *ripA* |  |
| MM31 | CAGGTCGACATCGATCTACTTTTGACCTTCTTGTTTTTCTATTCTTGG | *ripA* |  |
| OCN424 | GGATCCCCCGGGGAATTC |  |  |
| OCN425 | CAATATTTCAAGCTATACCAAGC |  |  |
|  |  |  |  |
| **Genes invalidation** | | | |
| MB207 | GCTCTTGAAGGGAACTATGTCAGATCGATTTGATAACATCATCCC | *ripB* deletion |  |
| MB208 | AGCGACGCCATCTATGTGTCGCCGTAATTCTTTCAATACCCGACC | *ripB* deletion |  |
| MB211 | GCTCTTGAAGGGAACTATGTCCAAGAATAGAAAAACAAGAAGGTC | *ripA* deletion |  |
| MB212 | AGCGACGCCATCTATGTGTCCCTCAAGCGAATCAAAGAGATCCTG | *ripA* deletion |  |
| MB307 | GCTCTTGAAGGGAACTATGTGAATTAGCTCAGAGAATTAATTTAACGG | insertion in *ripB* |  |
| MB308 | AGCGACGCCATCTATGTGTCGCTCTACTTAAACCATTTAACAGCCG | insertion in *ripB* |  |
| MB309 | GCTCTTGAAGGGAACTATGTGTGTCGAATGGTTATGCATGGACGCG | insertion in *ripA* |  |
| MB310 | AGCGACGCCATCTATGTGTCCATTGTCTCTCTTTTTTCTAGACGTTC | insertion in *ripA* |  |
| MP170 | GACACATAGATGGCGTCGCT | *aphA-3* or a*ad9* amplification |  |
| MP173 | ACATAGTTCCCTTCAAGAGC | *aphA-3* or *aad9* amplification |  |
|  |  |  |  |
| **CEP_lac_-X constructions** | | | |
| MB291 | CGATAGCTTTAACATTAGCCTTCTTATCAT |  |  |
| MB293 | GAATTCTCAGGCGGTCAACGTCAACGTATC |  |  |
| MB294 | ATGTACACCTCCTTAAGCTTAATTGTTATCCGCTC |  |  |
| MB295 | GGATCCGTTTGATTTTTAATGGATAATGTGATATAATC |  |  |
| MB296 | GAGCGGATAACAATTAAGCTTAAGGAGGTGTACATATGTTGAATCAGGATCTCTTTGATTCGCTTG |  |  |
| MB297 | GATTATATCACATTATCCATTAAAAATCAAACGGATCC TTATAAATTATTTCTTTTGTACAAAGGGATG |  |  |
| MB298 | GATTATATCACATTATCCATTAAAAATCAAACGGATCCCTACTTTTGACCTTCTTGTTTTTCTATTCTTG |  |  |
| MB299 | GAGCGGATAACAATTAAGCTTAAGGAGGTGTACATATGTTTAATGGTCGGGTATTGAAAGAATTACG |  |  |
| MB585 | tcaggatctggtggagaagcagcagctaaagctggaatgttgaatcaggatctctttgattcgcttgagg |  |  |
| MB586 | cccacaaaatcttcaagtgtaaaaacCATatgtacacctccttaagcttaattgttatccgc |  |  |
|  |  |  |  |
| **CEPII_lac_-X constructions** | | | |
| MM56 | GGACGAAATTTGTCCTTTTTATTGACCAGATTCCAAACG |  |  |
| MM57 | GCTTCTCGAGGGTACCTTACTACTTTTGACCTTCTTGTTTTTCTATTC |  |  |
| MM58 | CGTTTGGAATCTGGTCAATAAAAAGGACAAATTTCGTCC |  |  |
| MM59 | GAATAGAAAAACAAGAAGGTCAAAAGTAGTAAGGTACCCTCGAGAAGC |  |  |
| MB594 | tccggaaccctcgagtagaatttcttcaaaaagtctataacctgtaaccaTatgtacacctccttaagcttaattgttatccgc |  |  |
| MB595 | Tagggatccgtttgatttttaatggataatgtg |  |  |
| MB596 | cacattatccattaaaaatcaaacggatccctAgtttatagttactctaaacaacatagatccatcagg |  |  |
| MB597 | Ctttttgaagaaattctactcgagggttccggaatgattcatttttcaattaataaaaatttatttctacaagcattaaatattac |  |  |
| MB598 | ctttgttaagcttctcgagggtaccttAttaatttgtacgaactggtgtaatgagctg |  |  |
| MB599 | Taaggtaccctcgagaagcttaacaaag |  |  |
| YA09 | CGTGACAGGTGGAGCTGG |  |  |
| YA14 | CCAAAGCTATGCGCCAAAGAGACC |  |  |
|  |  |  |  |
| **CRISPRi depletion** | | | |
| OIM134 | GTTTAAGAGCTATGCTGGAAACAGC | plasmid amplification |  |
| OIM135 | TATAGTTATTATACCAGGGGGACAGTGC | plasmid amplification |  |
| MM54 | GCATAGCTCTTAAACGTAGTTATTGAACAAACAAGTATAGTTATTATACC | *clpP* |  |
| MM55 | GGTATAATAACTATACTTGTTTGTTCAATAACTACGTTTAAGAGCTATGC | *clpP* |  |
| MB551 | GCATAGCTCTTAAACAAACCAATTGAACTCCTCCATATAGTTATTATACC | *clpX* |  |
| MB552 | GGTATAATAACTATATGGAGGAGTTCAATTGGTTTGTTTAAGAGCTATGC | *clpX* |  |
|  |  |  |  |
| **Error prone PCR** |  |  |  |
| MB313 | CGCGTGTTAAATATATCCCTGCCG | *DnaN* locus amplification |  |
| MB314 | TGCCATCTTTTCTACACGCGCA | *DnaN* locus amplification |  |
