# Supplementary material for: A toxin/antitoxin system targeting the replication sliding-clamp induces competence in Streptococcus pneumoniae
Source: PLoS Genet. 2025 Dec 29;21(12):e1011863. doi: 10.1371/journal.pgen.1011863 (PMC12795458; doi:10.1371/journal.pgen.1011863)
Supplement: Attachment — Submitted filename: Review for PGENETICS-D-25-00966.pdf [file pgen.1011863.s010.pdf]

In this paper, the authors show that, in S. pneumoniae, the competence development caused by depletion of the ATPase ClpX (part of the Clp protease complex) is directly linked to the RipA toxin activity. The study convincingly establishes RipA as a toxin whose activity depends on interaction with DnaN and is counteracted by RipB, but the mechanistic link between RipA-DnaN interaction, competence, and toxicity remains unclear. The role of ClpX and RipB in blocking RipA activity is not sufficiently explored, and the experiments do not demonstrate whether RipA-induced competence occurs under natural conditions. In addition, many figures lack replication data or proper presentation of replicates, and the manuscript is sometimes difficult to follow due to inconsistent nomenclature and incomplete construct descriptions.

Major comment / molecular mechanism of RipA-RipB-ClpX-DnaN interplay

The authors show that depletion of ClpX is toxic and induces competence if the RipA toxin is present. They further show that RipB is the anti-toxin of RipA, that overexpressing RipA alone leads to cell toxicity and competence induction, and that producing RipB and certain punctual mutations in the sliding clamp DnaN cancel both these RipA-induced phenotypes. They convincingly show a direct interaction between RipA and DnaN, and that this interaction is crucial to cause both RipA-induced phenotypes. However, it is not clear how the RipA-DnaN interaction cause competence and toxicity. Is replication blocked or slowed down? Is there a direct effect on comCDE or comAB basal expression?

While it is interesting that the RipA toxin can interact directly with DnaN, it is not clear how both ClpX and RipB are necessary to block RipA activity. Is there a direct interaction? Or is there some modifications or targeted degradation? The hypotheses proposed by the authors are not developed or explained enough.

Major comment / role of RipA-RipB-ClpX during natural competence development

The experiments presented here do not show if the RipA-dependent competence induction happens in real life, for example in response to stress (which is what is assessed by the authors: L93-97, L300-302, L307-308). The induction of competence in cells neighboring those that have, by mistake or due to stress, expressed a bit more RipA, would be quite interesting. It could be another way in which heterogeneity could trigger a full-scale response at the population level. However, none of the experiments assembled for this paper are done in natural condition. They mixed RipA producing cells (which they know develop competence) with cells that can respond to external CSP and indeed show that the latter develop competence. This is a classical mechanism, even the same team published recently a model of competence propagation/induction in heterogenous population (https://doi.org/10.1038/s41467-024-49853-2). Using time lapses and fluorescence microscopy, the authors could have shown that (1) RipA (and/or ClpX and/or RipB) production is heterogenous, (2) cells with high RipA production are dying and surrounded by cells becoming competent. Or at least it could be tested if RipA (and/or ClpX and/or RipB) production are modified by stress (induction of RipA?, repression of ClpX/RipB?). Maybe some already produced dataset (RNAseq? Mass spec?) can be checked?

Major comment / lack of replication or missing data from replicates

A lot of experimental replicates are not shown. The authors often write “only a single data set, representative of at least three independent experiments carried out on different days, is presented” or sometimes do not mention any replication:

→ fig 1A&B, 6, 7D: if the data from replicates are too noisy to be compiled on a single graph (curve with error bars), the results of the other independent experiments should be shown as supplemental fig (also no mention of any replicates are noted for fig 6)

→ fig 2, 7C: no mention of any replicates

→ fig 3B&C, 4C: only results from a “representative experiment” are shown even though it would be easy to include the results of multiple replicates in the histogram representation of the platings

→ fig 3D, 4A, 7B: there are error bars but it is not noted how many replicates were done and individual data points should be shown

→ fig 5: results for negative controls and replicates should be presented as supplemental fig

Comment / clarity, nomenclature

The paper is quite hard to follow in some places because the different expression platforms and strains are often not described in the text or figure legends, so the reader has to go back and forth between the text / method / genotype table. Even so, multiple inconsistencies are present in the descriptions of certain constructions and in the nomenclature used, and a lot of constructs/genetic tools are not described.

For example, L106-107 states "Briefly, the defective Streptococcus pyogenes Cas9 (dCas9) controlled by an IPTG-dependent promoter was integrated into the S. pneumoniae chromosome together with the lacI gene and a single-guide RNA (sgRNA), targeting clpX or clpP, under the control of constitutive promoters." The legend of fig1 states that these are strains R4993 and R5203. Then, we have to go to the table S1, and see the genotype "*ComC::luc, PF6-lacI, Plac dCas9, p3-sgRNA-clpX*" and "*ComC::luc, PF6-lacI, Plac dCas9, p3-sgRNA-clpP*". The nomenclature is messy:

- not everything should be in italics,
- *ComC* should not start with a capital letter,
- :: usually means insertion but here the strains still have a functional *comC*, so it should be "*comC*::*luc*, *comC*", also, apparently the *luc* gene in this construction is associated with an erythromycin resistance gene, so it should be noted "*comC*::[*luc*, *xxx*], *comC*" with *xxx* being the gene conferring the resistance to erythromycin
- the 3 cassettes (for *lacI*, *dCas9* and *sgRNA*) should have their insertion site and their antibiotic resistance gene if there is one (L357-364 are not clear on how these strains were created)
- nomenclature should be PF6-*lacI;* P_{lac}-*dCas9* and P3-*sgRNA-clpX* (promoters in capital, not italics; an hyphen should be present between promoter and genes; inducible promoter are normally written with subscript)
- antibiotic resistance should be noted after the genotype (for ex AmpR), but the actual antibiotic resistance gene should be included in the genotype (for ex *bla*)
- the genotype of R5198 is not understandable (and the construction of this strain is not described)

For the expression platforms: a uniform nomenclature should be used. Sometimes, the auhors write CEPlac spr1630, or CEPlac-spr1630, or CEPlac-ripA, or CEPlac::ripA, or pCEPlac::ripA, or CEP_{lac}-ripA, also used: PcepII-lac, $CepII_{lac}$, CEP_{lac}. It would be easier to follow if it was consistent. I would propose to fully describe the platforms in the mat&meth, and to use a proper nomenclature. It is not written what exactly are on these expression platforms apart from an IPTG-inducible promoter followed by the gene of interest: lacI repressor? Antibiotique resistance ? In the figures, P_{lac}-*ripA* should suffice (CEP I or II is useless). In the genotypes table something like this could be used: *ami*::CEP[lacI, P_{lac}-*ripA*, *nptII*]; *cpsN*::CEPII[lacI, P_{lac}-*ripA*, *nptII*].

Minor comments:

L72-73: it is not described which "2 pathways" the authors are referring to. The readers are not supposed to read reference 20 (doi:10.1371/journal.pgen.1010946) to understand the sentence. Also if a "pathway" refers to a molecular mechanism that explains why a certain stress triggers competence, then at least 2 more pathways were described: doi:10.1128/mBio.00071-11, doi:10.1016/j.cell.2014.01.068.

L93 : Renaming the TA genes from numbers to a meaningful acronym is good. However it should be then be used consistently throughout the manuscript. In the mat&meth, L343-344, the numbers are used, even though the platforms are written with the names in the genotypes presented in table S1. Fig 2A is the only one in my opinion that need to show both the numbers and the names (as well as the nucleotides number to show the chromosomal locus).

L117: "Fig 1B" -> it would be helping to write "compare 1B to 1A", same L121

L123-125: I disagree. As only one experiment is shown and there is no standard deviation on the growth curves, you cannot conclude on the tiny difference between the red curves shown in bottom right in 1A and 1B.

L129-131: it is weird to explain PF6 here as it was already present in the strains used in the paragraph above (for fig1)

L138: “impairs this growth defect” -> cancel a defect seems more appropriate
L216-218: not clear
L220: “RipA-exposed cell” -> RipA-producing would be better
L227: “RipA stressed cell” -> RipA-producing would be better
L238-240: same as comment for L123-125
L253: cite fig2&3
L257: “ectopic expression of ripA was impaired” -> activity was impaired not expression
L265-266: where was that shown?
L278: “sliding Clamp” -> clamp
L294: writing the web address seems strange
L322: “plated on CAT”-> is it not “in CAT” instead?
L325: “the selection”-> delete “the”
L329: “storage”-> stored
L336: put the tables numbers
L341: “and sequenced (Eurofins genomics).”-> and the transformed locus was sequenced (Eurofins genomics).
L342-355: It is said that R3833 was used as template for PCR, this strain should be in Table S1. Transformation with the ami platform is followed by kanamycin selection, and the cps platform is described as a transfer of the ami platform to a new locus. So I suppose it also bears a kanR gene. If so, how was the R5259 strain was created, as it contains both platforms? Was the resistance gene changed? Or was it a double transformation with kan selection?
L353: R4631 is not described and not in table S1
L357-359: addgene numbers and publication of reference for the 3 plasmids used
L363-364: unclear, no selection is mentioned nor the insertion loci
L368: ref for plasmid pR414? Description? The genotype should be written in table S1
L384: how are these “potential suppressor clone” chosen or selected?
L385-386: how were the DnaN mutants selected after transformation in R5086? It is noted “as previously described” without any reference. If “as described above” was meant then what selection was used? Are the DnaN mutations linked to a resistance gene?
L385: R5086 is a comC0 strain but all DnaN mutants in table S1 are in a comC::luc background, is there a mistake somewhere?
L393: R1501 is not described, there are no reference, and it is not noted in table s1
L395: replace spr1630 by ripA
L606-608: unclear, see comments above on CEP nomenclature
L615: “see mat&meth” it would be easier if the explanation (MgCl2 reduces fidelity) was in the legend, or you could write mutagenized+ / mutagenized++
L621: indicate pdb number
L640: indicate the strength of the mutation, next to the strain number
L648: is the strain used R5139 or R5204?
L654: why suddenly PcomCDE::luc ? it should be “comC::luc, comC” like everywhere else
L658: what transfo test was done? At what time?
