# Supplementary material for: A toxin/antitoxin system targeting the replication sliding-clamp induces competence in Streptococcus pneumoniae
Source: PLoS Genet. 2025 Dec 29;21(12):e1011863. doi: 10.1371/journal.pgen.1011863 (PMC12795458; doi:10.1371/journal.pgen.1011863)
Supplement: Attachment — Submitted filename: Point-by-Point Response to Reviewers 25-12-10.docx [file pgen.1011863.s011.docx]

**Reviewer #1:**

1. The conclusion that the toxin RipA induces competence activation and growth arrest is based on its targeting of DnaN (the sliding clamp), thereby disrupting DNA replication. While this interpretation is reasonable, no direct evidence demonstrates actual inhibition of bacterial DNA replication. This point becomes more doubtful given the absence of any sequence or structural homology between RipA and SocB, the first toxin reported to target the sliding clamp in Caulobacter crescentus (line 285), nor between their respective antitoxins (line 293).

We may have been somewhat unclear in our initial wording by highlighting only the differences between RipA and SocB without emphasizing their functional similarities. Nevertheless, the two proteins are indeed significantly different. Therefore, to strengthen our demonstration of the impact of RipA on replication, we performed several additional experiments, which we have now incorporated into the manuscript.

To support the *in vivo* interaction, we conducted a split-luciferase assay in *S. pneumoniae*, which confirms the interaction between RipA and DnaN and shows that this interaction is altered in the DnaN mutants (Fig 5B).

We also monitored the behavior of the replication fork by fluorescence microscopy, using fluorescent DnaX—the clamp loader commonly used for this purpose in *S. pneumoniae*. We observed that a fraction of the population expressing RipA loses the DnaX focus, and among the cells that retain a detectable focus, nearly forty percent display an aberrant (polar) localization of the fluorescent DnaX. Overall, after 90 minutes, about fifty per cent of the cells exhibited abnormal behavior, compared to cells that did not produce RipA (Figure S7).

We also examined the basal transcription levels of the competence genes *comC* and *comA*, as well as that of a tRNA gene located near *comC* but not induced during competence. We observed that increasing RipA production led to a rise in the basal transcription level of *comC* (Fig 2C), as well as that of the neighboring tRNA (Fig S3A), whereas *comA* was only marginally affected (Fig S3A). These observations are consistent with previous findings showing that “replication damage” can enhance transcription of origin-proximal genes, likely through gene dosage effects (Slager J, Kjos M, Attaiech L, Veening J-W. Antibiotic-induced replication stress triggers bacterial competence by increasing gene dosage near the origin. Cell).

This has also enabled us to enhance Figure 8.

The authors demonstrated that RipA-triggered DNA replication arrest induces bacteria to become competent. This raises an interesting question: does the activation of competence alleviate the toxic effects of RipA itself?

This is indeed a very interesting question. At present, however, our available data do not allow us to address it conclusively. While our current investigations do not point in this direction, we recognize the relevance of this hypothesis. Clarifying the biological relationship between competence and this toxin–antitoxin system will require dedicated future studies, as this question cannot be resolved with the data currently available. We believe that identifying the specific stress or stresses that activate this system will be a key aspect of future work.

2. To further demonstrated RipAB belongs to a TA system, it’s better to provide evidence of co-transcription of these two genes.

We acknowledge that we did not sufficiently highlight this point, which is already well established in the literature through RNA-seq studies (*slager et* al., 2019) and is accessible via the PneumoBrowse database. We have now addressed this in the revised manuscript (L132).

3. Line 259: Regarding the relationship between ClpX and RipAB, is it possible that the basal expression level of RipB is insufficient to counteract the toxicity of RipA? If so, overexpressing RipB might eliminate the dependence on RipA for ClpX function.

Following your comment, we performed this experiment, and indeed, RipB overexpression abolishes the dependence on ClpX (Fig S3B). This result is particularly interesting, as it establishes a link between ClpX and the entire TA system. For this reason, we have also modified Figure 8 to depict ClpX as a potential catalyst of RipA inactivation.

However, we prefer to remain cautious in drawing definitive conclusions at this stage, as a comprehensive characterization of this TA system—representative of a new TA subclass—will require additional investigations. These studies are part of an ongoing four-year collaborative research project.

4. Fig. 4B: is there any clear annotation of the three domains of DnaN, which may indicate their functions?

Indeed, DnaN is composed of a repetition of three structurally analogous, although not identical, domains, and it is this subdivision that is typically highlighted. Previous studies have shown that these domains are partially specialized. For example, the third domain mediates interactions between DnaN and several of its protein partners, whereas the second domain is involved in binding the polymerase. However, this domain-based division remains somewhat difficult to interpret functionally, which is why we did not place too much emphasis on the specific roles of the other two domains.

5. Is there any clue that reveals the interaction mechanism between RipA and DnaN by predicted structural analysis, particularly RipA with the five different amino acids of DnaN (line 164).

Yes, we attempted to model the interaction between RipA and DnaN using AlphaFold3. The model suggested an interaction involving, among others, residues 182, 374, 376, and 248 of DnaN, which is consistent with our genetic analysis. However, the predicted interaction surface with RipA appears to be more extensive, and we are unable to validate this experimentally through simple mutagenesis in *dnaN* due to its essential nature. Moreover, although the interaction scores support the interaction, they are not sufficiently robust to be considered definitive. For these reasons, we have initiated a crystallography project on the RipA–DnaN complex with our collaborators, which will require dedicated work.

We would therefore prefer to highlight the experimental evidence for this interaction by adding a split-luciferase assay (Fig S3B) to further support our conclusions, rather than including a modeling figure at this stage. However, if you consider it absolutely necessary, we could include this preliminary model.

6. The authors claim that bacterial multiplication was affected after 90 minutes of IPTG exposure, a conclusion based solely on the microscopy data presented in Fig. 7A. Statistical analysis of multiple microscopy fields through quantification of bacterial cell counts over the course of IPTG exposure would be necessary to support this assertion.

We have added two new microscopy panels originating from two independent experiments (Fig S7A), as well as cell counts performed across multiple fields from each experiment (Fig S7B).

7. Lines 219 and Fig. 7C: Why did cells producing RipA with IPTG treatment for more than 60 minutes exhibit more rapid and higher competence induction in response to synthetic CSP? The induction of RipA by IPTG appears to enable at least a subset of pneumococcal cells to become competent. However, it is well-established that bacterial cells enter a recovery phase following competence, which seems inconsistent with the authors’ observation.

Initially, this experiment was designed to address a single question: were the cells losing viability under the action of RipA still metabolically active and capable of responding to CSP? This was unequivocally the case. However, we agree that the observation of a stronger and more prolonged response is intriguing. We hypothesized that this phenomenon could result from the effect of RipA on replication. To support this hypothesis, we examined the response capacity of the same cells subjected to a replicative stress induced by HPUra (0.125 µM), a base analogue that targets replication and triggers overtranscription of genes located at the origin of replication through a mechanism that increases the ori/ter ratio. Strikingly, we observed the same behavior in cells treated with HPUra or producing RipA (Fig 7C).

This finding is consistent with the observations presented in Figures 2 and S3 and strongly supports the hypothesis that RipA affects replication by inducing an overproduction of *comCDE* RNA through a gene-dosage mechanism. These points have been incorporated into the Results and the Discussion.

9. Line 259: ‘dependant’ should be ‘dependent’.

Corrected.

10. Line 288: ‘supressing’ should be ‘suppressing’.

Corrected

**Reviewer #3**: MS # PGenetics-D-25-00966, by Maziero et al. brings to light a new toxin/antitoxin system in S. pneumoniae that links the toxin component to competence for genetic transformation and stress responses. The tandem gene pair, which was noted as affecting the essentiality of ClpX nearly two decades ago, but with unknown functions, is now designated as RipA,RipB (for replication interfering protein). Thorough genetic and biochemical dissection in this manuscript now reveals, surprisingly, that the molecular target of the toxin component is DnaN, the sliding clamp of DNA replication.

A model of expository clarity, the submitted manuscript needs little further revision. However, careful review, perhaps best done by a first-time reader, would be rewarded by unearthing a few remaining typos.

We thank Reviewer 3. We corrected remaining typos.

**Reviewer #2: my review is in the attached file**

In this paper, the authors show that, in S. pneumoniae, the competence development caused by depletion of the ATPase ClpX (part of the Clp protease complex) is directly linked to the RipA toxin activity. The study convincingly establishes RipA as a toxin whose activity depends on interaction with DnaN and is counteracted by RipB, but the mechanistic link between RipA-DnaN interaction, competence, and toxicity remains unclear. The role of ClpX and RipB in blocking RipA activity is not sufficiently explored, and the experiments do not demonstrate whether RipA-induced competence occurs under natural conditions. In addition, many figures lack replication data or proper presentation of replicates, and the manuscript is sometimes difficult to follow due to inconsistent nomenclature and incomplete construct descriptions.

Major comment / molecular mechanism of RipA-RipB-ClpX-DnaN interplay

The authors show that depletion of ClpX is toxic and induces competence if the RipA toxin is present. They further show that RipB is the anti-toxin of RipA, that overexpressing RipA alone leads to cell toxicity and competence induction, and that producing RipB and certain punctual mutations in the sliding clamp DnaN cancel both these RipA-induced phenotypes. They convincingly show a direct interaction between RipA and DnaN, and that this interaction is crucial to cause both RipA-induced phenotypes. However, it is not clear how the RipA-DnaN interaction cause competence and toxicity.

Is replication blocked or slowed down? Is there a direct effect on comCDE or comAB basal

expression?

We have added several experiments to address these two questions.

To support the *in vivo* interaction, we conducted a split-luciferase assay in *S. pneumoniae*, which confirms the interaction between RipA and DnaN and shows that this interaction is altered in the DnaN mutants (Fig 5).

We also monitored the behavior of the replication fork by fluorescence microscopy, using fluorescent DnaX—the clamp loader commonly used for this purpose in *S. pneumoniae*. We observed that a fraction of the population expressing RipA loses the DnaX focus, and among the cells that retain a detectable focus, nearly forty percent display an aberrant (polar) localization of the fluorescent DnaX. Overall, after 90 minutes, about 40 per cent of the cells exhibited abnormal behaviour with regard to the localisation of DnaX-YFP, compared to cells that did not produce RipA (Fig S7).

We also examined the basal transcription levels of the competence genes *comC* and *comA*, as well as that of a tRNA gene located near *comC* but not induced during competence. We observed that increasing RipA production led to a rise in the basal transcription level of *comC* (Fig 2C), as well as that of the neighboring tRNA (Fig S3A), whereas *comA* was only marginally affected (Fig S3A) as *comA* appears to lie at the boundary of the genes that are overinduced under replicative stress (Slager J, Kjos M, Attaiech L, Veening J-W. Antibiotic-induced replication stress triggers bacterial competence by increasing gene dosage near the origin. Cell).

Finally, we also tested the effect of a DNA polymerase inhibitor (HPUra) and observed that the transcriptional phenotypes of the *comCDE* operon were similar (Fig 7C).

These observations are consistent with previous findings showing that “replication damages” can enhance transcription of origin-proximal genes, likely through gene dosage effects (Slager J, Kjos M, Attaiech L, Veening J-W. Antibiotic-induced replication stress triggers bacterial competence by increasing gene dosage near the origin. Cell). The addition of these supplementary data allowed us to refine Fig 8.

While it is interesting that the RipA toxin can interact directly with DnaN, it is not clear how both ClpX and RipB are necessary to block RipA activity. Is there a direct interaction? Or is there some modifications or targeted degradation? The hypotheses proposed by the authors are not developed or explained enough.

Following the suggestion of Reviewer 2, we overexpressed RipB (the antitoxin) to determine whether this could bypass the requirement for ClpX. This was indeed the case, providing strong evidence for a functional link between RipB and ClpX (Fig S3B). These results allow us to propose the hypothesis that ClpX acts as a catalyst for the neutralization of RipA by RipB. In the Discussion, we have added new elements and elaborated on the most plausible hypotheses (Fig 8).

However, given the diversity of molecular mechanisms underlying toxin–antitoxin systems and their autoregulatory processes, a detailed mechanistic understanding will require dedicated work. Indeed, this is the focus of an ongoing four-year research project

Major comment / role of RipA-RipB-ClpX during natural competence development

The experiments presented here do not show if the RipA-dependent competence induction happens in real life, for example in response to stress (which is what is assessed by the authors: L93-97, L300-302, L307-308). The induction of competence in cells neighboring those that have, by mistake or due to stress, expressed a bit more RipA, would be quite interesting. It could be another way in which heterogeneity could trigger a full-scale response at the population level. However, none of the experiments assembled for this paper are done in natural condition.

Indeed, approximating ‘natural conditions’ would be ideal, even though such conditions are always relative in the context of fundamental research. These studies would nevertheless be greatly facilitated by the identification of the specific stress that triggers the imbalance of the ClpX–RipB–RipA system. We tested several known stresses in a Δ*ripA* or ΔripAB context; unfortunately, the specific ‘signal’ remains elusive for now, but we remain hopeful to identify it in the future.

Nevertheless, to approximate more “physiological conditions”, we examined the behavior of the Δ*ripA* and Δ*ripAB* mutants under conditions that are limiting for competence induction. The results are presented Fig S2B. We observed that both mutants displayed a lower frequency of competence induction compared to the wild type, which we interpret as a deficit in integrating inducing signals under these culture conditions. This suggests that the RipAB system contributes to competence induction under certain conditions. However, working in these conditions is challenging, as the signal(s) relayed by the RipAB system are intertwined with numerous other inducing signals.

They mixed RipA producing cells (which they know develop competence) with cells that can respond to external CSP and indeed show that the latter develop competence. This is a classical mechanism, even the same team published recently a model of competence propagation/induction in heterogenous population (<https://doi.org/10.1038/s41467-024-49853-2>).

The aim of the experiments presented in Fig 7 is not to characterize the mechanism of signal propagation, which has indeed been extensively deciphered in various studies from different laboratories. Rather, these experiments aim to determine whether cells in a highly unfavourable physiological state are still capable of responding and, importantly, of transmitting the signal to other cells.

Using time lapses and fluorescence microscopy, the authors could have shown that (1) RipA (and/or ClpX and/or RipB) production is heterogenous, (2) cells with high RipA production are dying and surrounded by cells becoming competent. Or at least it could be tested if RipA (and/or ClpX and/or RipB) production are modified by stress (induction of RipA?, repression of ClpX/RipB?). Maybe some already produced dataset (RNAseq? Mass spec?) can be checked?

All of these experiments are indeed interesting, but they are difficult to perform in a context where the stress sensed or mediated by RipAB remains unknown. As mentioned above, multiple other stresses are perceived by other signalling pathways, making it challenging to distinguish their effects under these conditions. Nevertheless, these experiments should be considered exploratory, providing initial insights that raise further questions and may, in the long term, contribute to a deeper understanding of how this TA system is integrated into the physiology of S. pneumoniae.

Major comment / lack of replication or missing data from replicates

A lot of experimental replicates are not shown. The authors often write “only a single data set, representative of at least three independent experiments carried out on different days, is presented” or sometimes do not mention any replication:

We agree, and for this reason, we have added all the requested data and clarified certain points. Overall, to improve the readability of the results, either the standard deviations and distribution of experimental points have been included in all figures, or the triplicates of the different experiments have been provided as supplementary figures, as requested.

→ fig 1A&B, 6, 7D: if the data from replicates are too noisy to be compiled on a single graph (curve with error bars), the results of the other independent experiments should be shown as supplemental fig (also no mention of any replicates are noted for fig 6)

modified

→ fig 2, 7C: no mention of any replicates

modified

→ fig 3B&C, 4C: only results from a “representative experiment” are shown even though it would be easy to include the results of multiple replicates in the histogram representation of the platings

modified

→ fig 3D, 4A, 7B: there are error bars but it is not noted how many replicates were done and

individual data points should be shown

modified

→ fig 5: results for negative controls and replicates should be presented as supplemental fig

Comment / clarity, nomenclature

modified

The paper is quite hard to follow in some places because the different expression platforms and strains are often not described in the text or figure legends, so the reader has to go back and forth between the text / method / genotype table. Even so, multiple inconsistencies are present in the descriptions of certain constructions and in the nomenclature used, and a lot of constructs/genetic tools are not described.

For example, L106-107 states “Briefly, the defective Streptococcus pyogenes Cas9 (dCas9) controlledby an IPTG-dependent promoter was integrated into the S. pneumoniae chromosome together with the lacI gene and a single-guide RNA (sgRNA), targeting clpX or clpP, under the control of constitutive promoters.” The legend of fig1 states that these are strains R4993 and R5203. Then, we have to go to the table S1, and see the genotype “ComC::luc, PF6-lacI, Plac dCas9, p3-sgRNA-clpX” and “ComC::luc,PF6-lacI, Plac dCas9, p3-sgRNA-clpP”. The nomenclature is messy:

- not everything should be in italics,

corrected

- ComC should not start with a capital letter,

corrected

- :: usually means insertion but here the strains still have a functional comC, so it should be

“comC::luc, comC”, also, apparently the luc gene in this construction is associated with an

erythromycin resistance gene, so it should be noted “comC::[luc, xxx], comC” with xxx being

the gene conferring the resistance to erythromycin

corrected

- the 3 cassettes (for lacI, dCas9 and sgRNA) should have their insertion site and their

antibiotic resistance gene if there is one (L357-364 are not clear on how these strains were

created)

added in material and methods

- nomenclature should be PF6-lacI; Plac-dCas9 and P3-sgRNA-clpX (promoters in capital, not

italics; an hyphen should be present between promoter and genes; inducible promoter are

normally written with subscript)

corrected

- antibiotic resistance should be noted after the genotype (for ex AmpR), but the actual

antibiotic resistance gene should be included in the genotype (for ex bla)

corrected in the genotypes table.

- the genotype of R5198 is not understandable (and the construction of this strain is not

described) For the expression platforms: a uniform nomenclature should be used. Sometimes, the auhors write CEPlac spr1630, or CEPlac-spr1630, or CEPlac-ripA, or CEPlac::ripA, or pCEPlac::ripA, or CEPlac-ripA, also used: PcepII-lac, CepIIlac, CEPlac. It would be easier to follow if it was consistent.

We homogenize to CEP_lac_-*ripA or* CEPII_lac_-*ripA*

I would propose to fully describe the platforms in the mat&meth, and to use a proper nomenclature. It is not written what exactly are on these expression platforms apart from an IPTG-inducible promoter followed by the gene of interest: lacI repressor? Antibiotique resistance ? In the figures, Plac-ripA should suffice (CEP I or II is useless). In the genotypes table something like this could be used: ami::CEP[lacI, Plac-ripA, nptII]; cpsN::CEPII[lacI, Plac-ripA, nptII].

We have included a general explanation of the platforms in the Materials and Methods section before detailing the specific constructions and bibliographic references.

However, we believe that including the chromosomal position of the platforms in the strain table would reduce the readability of the genotypes. We therefore prefer to detail this information in the Materials and Methods section.

**Minor comments**:

L72-73: it is not described which “2 pathways” the authors are referring to. The readers are not

supposed to read reference 20 (doi:10.1371/journal.pgen.1010946) to understand the sentence. Also

if a “pathway” refers to a molecular mechanism that explains why a certain stress triggers

competence, then at least 2 more pathways were described: doi:10.1128/mBio.00071-11,

doi:10.1016/j.cell.2014.01.068.

We agree that these references belong to the preceding sentence, and we have therefore simplified the wording accordingly. “Nonetheless, it remains evident that additional pathways have yet to be identified, and that some of the pathways currently known are still insufficiently characterized at the molecular level.” (lines 72-74)

L93 : Renaming the TA genes from numbers to a meaningful acronym is good. However it should be then be used consistently throughout the manuscript. In the mat&meth, L343-344, the numbers are used, even though the platforms are written with the names in the genotypes presented in table S1. Fig 2A is the only one in my opinion that need to show both the numbers and the names (as well as the nucleotides number to show the chromosomal locus).

We agreed. We modified accordingly

L117: “Fig 1B” -> it would be helping to write “compare 1B to 1A”, same L121

We agreed. We modified.

L123-125: I disagree. As only one experiment is shown and there is no standard deviation on the growth curves, you cannot conclude on the tiny difference between the red curves shown in bottom right in 1A and 1B.

The term may indeed be too strong. However, the addition of the growth curves with a linear y-axis (Figure S2B) reveals a trend toward improved growth. Although this is not a critical point for the rest of the manuscript, we would like to retain this observation, as we cannot exclude the possibility that ClpP plays even a minor role in regulating RipA activity. We therefore propose to soften the statement by using 'slightly'.

L129-131: it is weird to explain PF6 here as it was already present in the strains used in the paragraph

above (for fig1)

We have deleted PF6 to simplify matters, retaining only the bibliographical reference.

L138: “impairs this growth defect” -> cancel a defect seems more appropriate

We change for “cancels”

L216-218: not clear

We reformulated

“Fig. 7B shows that most cells lost the ability to form colonies after 45 minutes of exposure. Yet, since competence develops around 120 minutes of RipA-induced toxicity (Fig. 2B), we examined whether these cells remain functionally active, at least regarding their competence induction profile. To test this, RipA-producing cells were exposed to IPTG for over 60 minutes and then treated with synthetic CSP to rapidly trigger competence. “

L220: “RipA-exposed cell” -> RipA-producing would be better

modified

L227: “RipA stressed cell” -> RipA-producing would be better

modified

L238-240: same as comment for L123-125

modified

L253: cite fig2&3

modified

L257: “ectopic expression of ripA was impaired” -> activity was impaired not expression

We completely agree. Corrected.

L265-266: where was that shown?

This is a mistake. It was just a hypothesis. We have modified the sentence to reflect this change. L321 “,DnaN could to be more sensitive to the action of RipA.”

L278: “sliding Clamp” -> clamp

Corrected.

L294: writing the web address seems strange

We substituted it with a reference.

L322: “plated on CAT”-> is it not “in CAT” instead?

Exact. As we inoculate the bacteria into the agar medium, we corrected it.

L325: “the selection”-> delete “the”

Corrected.

L329: “storage”-> stored

Corrected.

L336: put the tables numbers

Corrected.

L341: “and sequenced (Eurofins genomics).”-> and the transformed locus was sequenced (Eurofins genomics).

Corrected.

L342-355: It is said that R3833 was used as template for PCR, this strain should be in Table S1.Transformation with the ami platform is followed by kanamycin selection, and the cps platform isdescribed as a transfer of the ami platform to a new locus. So I suppose it also bears a kanR gene. Ifso, how was the R5259 strain was created, as it contains both platforms? Was the resistance gene changed? Or was it a double transformation with kan selection?

We agree that the description was confusing because it did not specify that the Cps platform carries erythromycin resistance. We have now added the previously undescribed strains to Table S1 and clarified that the Cps platform contains the erythromycin resistance gene (R4631). Furthermore, the harmonization of the nomenclature you suggested above will also help improve the clarity of the strain construction

L353: R4631 is not described and not in table S1

Added to Table S1.

L357-359: addgene numbers and publication of reference for the 3 plasmids used

added

L363-364: unclear, no selection is mentioned nor the insertion loci

We clarified all the construction process.

L368: ref for plasmid pR414? Description? The genotype should be written in table S1

We did not express this clearly. This construct has been established in the laboratory for more than twenty years and has been used many times before. However, we agree that the wording in the manuscript could suggest that it was specifically generated for this study. We have therefore clarified this point.

” To monitor *comCDE* operon expression, we used a previously established transcriptional fusion [57]. Briefly, a fragment of the S. pneumoniae *comCDE* promoter (HindIII–BamHI) was inserted upstream of the luc gene in a non-replicative plasmid carrying erythromycin resistance. Homology-dependent integration of this recombinant plasmid into the pneumococcal chromosome was then selected using erythromycin.”

L384: how are these “potential suppressor clone” chosen or selected?

They were selected by the addition of 20µM of IPTG (production of RipA). We add “selected” to clarify

L385-386: how were the DnaN mutants selected after transformation in R5086? It is noted “as previously described” without any reference. If “as described above” was meant then what selection was used? Are the DnaN mutations linked to a resistance gene?

We added “as described above (selection with 20µM IPTG)” (Line 460)

L385: R5086 is a comC0 strain but all DnaN mutants in table S1 are in a comC::luc background, is there a mistake somewhere?

No, the mutants were initially isolated in a comC0 background, and many tests were performed in this context. However, it seemed more logical to transfer them into a wild-type genetic background to repeat all the experiments. In any case, no differences in phenotype were observed depending on the presence or absence of *comC* for any of the RipA toxicity assays.

L393: R1501 is not described, there are no reference, and it is not noted in table s1

Added.

L395: replace spr1630 by ripA

corrected

L606-608: unclear, see comments above on CEP nomenclature

corrected

L615: “see mat&meth” it would be easier if the explanation (MgCl2 reduces fidelity) was in the

legend, or you could write mutagenized+ / mutagenized++

Modified line 775 “PCR fragments amplified from dnaN locus either without or with 25 µM of MgCl2 to reduce polymerase fidelity”

L621: indicate pdb number

Added

L640: indicate the strength of the mutation, next to the strain number

Added

L648: is the strain used R5139 or R5204?

It was R5139. The experiment was repeated to gain a deeper understanding of the phenomenon, and accordingly, the figure legend has been thoroughly updated.

L654: why suddenly PcomCDE::luc ? it should be “comC::luc, comC” like everywhere else

corrected

L658: what transfo test was done? At what time?

We added in legend of Fig 7 : “PCR DNA conferring streptomycin resistance was added at T0. The cells were then collected at T100 and plated on dishes containing erythromycin and streptomycin.”
